# Dexmedetomidine Analgesia Effects in Patients Undergoing Dental Implant Surgery and Its Impact on Postoperative Inflammatory and Oxidative Stress

**DOI:** 10.1155/2015/186736

**Published:** 2015-06-15

**Authors:** Sisi Li, Yang Yang, Cong Yu, Ying Yao, Yujia Wu, Lian Qian, Chi Wai Cheung

**Affiliations:** ^1^Department of Anesthesia, Chongqing Key Laboratory for Oral Diseases and Biomedical Sciences, Stomatology Hospital of Chongqing Medical University, No. 426 Songshibeilu, Yubei District, Chongqing 401146, China; ^2^Duke-NUS Graduate Medical School Singapore, Singapore 169857; ^3^Department of Anesthesiology, The University of Hong Kong, Hong Kong

## Abstract

The aim of the study was to determine whether or not dexmedetomidine- (DEX-) based intravenous infusion in dental implantation can provide better sedation and postoperative analgesia via suppressing postoperative inflammation and oxidative stress. Sixty patients were randomly assigned to receive either DEX (group D) or midazolam (group M). Recorded variables were vital sign (SBP/HR/RPP/SpO_2_/RR), visual analogue scale (VAS) pain scores, and observer's assessment of alertness/sedation scale (OAAS) scores. The plasma levels of interleukin-6 (IL-6), tumor necrosis factor alpha (TNF-*α*), antioxidant superoxide dismutase (SOD), and the lipid peroxidation product malondialdehyde (MDA) were detected at baseline and after 2, 4, and 24 h of drug administration. The VAS pain scores and OAAS scores were significantly lower for patients in group D compared to group M. The plasma levels of TNF-*α*, IL-6, and MDA were significantly lower in group D patients than those in group M at 2 h and 4 h. In group M, SOD levels decreased as compared to group D at 2 h and 4 h. The plasma levels of TNF-*α*, IL-6, and MDA were positively correlated with VAS pain scores while SOD negatively correlated with VAS pain scores. Therefore, DEX appears to provide better sedation during office-based artificial tooth implantation. DEX offers better postoperative analgesia via anti-inflammatory and antioxidation pathway.

## 1. Introduction

Dental implants are considered one of the most common and popular treatment options for edentulous patients in modern dentistry. However, dental implantation remains significantly associated with pain and high levels of anxiety [1, 2]. Implant surgery requires bone preparation, sometimes flap and bone graft treatment. This usually results in tissue ischemia [3] and acute inflammation [4, 5] with concomitant increase in oxidative stress. Mild, even severe pain following implant surgery is extremely to be expected [6]. Implant surgery causes tissue injury, resulting in pain hypersensitivity, as a result of peripheral sensitization (sensitization of primary sensory neurons) [7, 8] and central sensitization (sensitization of spinal cord and brain neurons) [9–11]. More and more dental patients choose general anaesthesia for comfortable and painless surgery. In these cases, the most widely used form is the combination of benzodiazepine with opioid [12, 13]. Our previous study indicated that the DEX/fentanyl regimen appears to be better than the traditional midazolam/fentanyl regimen in terms of intraoperative arousal, patient-surgeon cooperation, postoperative analgesia, and surgeon satisfaction in office-based unilateral impacted tooth extraction [14]. However, the effects and detailed mechanisms of postoperative analgesia effect of DEX in patients undergoing dental implantation surgery have yet to be revealed.

DEX, a selective agonist of *α*_2_-adrenergic receptor, selectively binds to presynaptic *α*_2_ adrenergic receptors norepinephrine release, resulting in a reduction of postsynaptic adrenergic activity [15]. DEX is a potent sedative agent and also provides analgesia and anxiolytic and sympatholytic effects and has minimal influence on respiratory physiology. Along with its beneficial effects, DEX was reported to exert potential anti-inflammatory and antioxidant effects. Previous studies revealed that DEX significantly decreased the levels of inflammatory cytokines during postpartum bleeding-induced multiple organ dysfunction syndrome in rats [16], in polymicrobial sepsis in mice [17], during cardiac surgery with cardiopulmonary bypass in human [18], and in lung injury in dogs [19] and laparoscopic cholecystectomy in human [20]. DEX also significantly decreased the levels of free radicals on ischemia-reperfusion injury of epigastric island flaps of rats [21] and ischemic rat hippocampus [22]. Our research chose proinflammatory IL-6, TNF-*α*, SOD, and MDA to reflect inflammatory and oxidation conditions in vivo.

It has been proposed that irrespective of the characteristic of the pain, whether it is sharp, dull, aching, burning, stabbing, numbing, or tingling, all pains arise from inflammation and the inflammatory response [23]. Our study aims to investigate the sedation and analgesia effect of DEX during and after implant surgery compared with midazolam and whether DEX offered better postoperative analgesia by regulating the inflammatory and oxidative stress.

## 2. Materials and Methods

### 2.1. Subjects and Study Protocol

Sixty patients enrolled in this project either have previously used DEX, midazolam, paracetamol, and other nonsteroidal anti-inflammatory drugs or had no known allergy to these drugs. All patients provided written informed consent. The patients were of American Society of Anesthesiology (ASA) physical status I or II, between 19 and 60 years old, and with mandibular teeth defect (33, 34, and 35 or 43, 44, and 45). The patients were to have 3 dental implants to be placed and flap and bone graft were to be performed during surgery. Patients were excluded if they had a clinical history or electrocardiographic evidence of heart block, ischemic heart disease, asthma, sleep apnea syndrome, impaired liver or renal function, known psychiatric illness, diabetes, facial pain, psychological problems, smoking history, or chronic use of sedative or analgesic drugs or opioids. Also excluded were those who refused to participate, were pregnant, or presented with preoperative inflammation at the site of surgery.

The 60 patients were randomly divided into two treatment groups using a computer-generated random list. Patients were infused either with midazolam and fentanyl (group M) or with DEX and fentanyl (group D). Each patient had an intravenous cannula inserted. Investigators who were not directly involved in the care of the patient prepared the infusions, while the dental surgeon, anesthetist, and the patients were blinded to the group allocation and drugs given.

Patients in group D received DEX (1.0 *μ*g/kg) and fentanyl (0.001 mg/kg) in 20 mL of normal saline for 10 min and then a continuous infusion of DEX (1.0 *μ*g/kg/h) until the end of the surgery. Patients in group M received midazolam (0.05 mg/kg) and fentanyl (0.001 mg/kg) in 20 mL of normal saline for 10 min, followed by a continuous infusion of midazolam (0.05 mg/kg/h) until the end of the surgery. Ten minutes after the start of the loading dose, local anesthesia was provided with 4% hydrochloric articaine and 1/100000 adrenaline, administered by qualified dental surgeons. Surgeons then performed the standard surgical procedure during which patients were provided with a mouth prop to help keep the mouth open when required. At the end of the operation, patients were kept in the recovery area 4 h after drug administration. Patients were prescribed one analgesic tablet containing 500 mg of paracetamol and then oral amoxicillin capsule 500 mg three times a day and ornidazole capsule 500 mg twice a day until 7 days after surgery.

### 2.2. Enzyme-Linked Immunosorbent Assay (ELISA)

Venous blood samples (3.0 mL each) were drawn at 0, 2, 4, and 24 hours after drug administration for the measurement of plasma cytokines. Plasma samples were immediately separated by centrifugation at 3,000 rpm for 10 min at 4°C and then divided into aliquots and stored at −80°C for subsequent assays by highly sensitive enzyme-linked immunosorbent assays (ELISA) kits to detect the proinflammatory cytokine (IL-6, TNF-*α*), antioxidant enzyme superoxide dismutase (SOD), and serum levels of lipid peroxidation product (MDA).

The production lot numbers and manufacturer of ELISA kits are SOD Human ELISA Kit (ab119694, abcam, UK); MDA Human ELISA Kit (E90597Hu, biorbyt, UK); TNF-*α* Human ELISA (BMS223/4CE, eBioscience, USA); Interleukin-6 Human ELISA Kit (501030-96, Cayman, USA).

### 2.3. Outcome Measures

All indices were recorded before initiating sedation (i.e., baseline) and then at 15 min intervals until 4 h after the start of drug infusion. Systolic blood pressure (SBP), heart rate (HR), rate-pressure product (RPP), breathing rate (RR), and saturation of pulse oxygen (SpO_2_) were recorded at 15 min intervals until 4 h after the start of drug infusion. Sedation levels were assessed using observer's assessment of alertness/sedation scale (OAAS). The patients evaluated their level of pain subjectively using a VAS ruler, with zero representing no pain and 10 the worst pain the patient had ever experienced.

### 2.4. Statistical Analyses

All variables were tested for normal distribution using the Shapiro-Wilk test. The data are expressed as the mean ± standard deviation (SD), median and interquartile range (IQR), or number. The OAAS and VAS scores were analyzed using the Kruskal-Wallis test. SpO_2_, HR, RR, and SBP values, age, weight, duration of surgery, number of dental implants, total volume of local anaesthetic used, TNF-*α*, IL-6, SOD, and MDA concentration were analyzed using the two-sample *t*-test. Gender was analyzed using the *χ* ^2^ test. The correlation between VAS and SOD, VAS and MDA, VAS and TNF-*α*, TNF-*α* and MDA, TNF-*α* and SOD was analyzed using Spearman rank correlation analysis. Statistical analyses were performed using the commercial software SPSS17.0 (SPSS, Institute, Chicago, IL, USA). *P* values of <0.05 were considered statistically significant.

## 3. Results

Sixty patients were recruited. The patient characteristics and operation data of both groups are shown in Table 1. There was no significant difference in demographic data, surgical characteristics, duration of operation, and total volume of local anaesthetic used between the two study groups. All patients have no preoperative inflammation at the site of surgery. There was also no difference in the overall preoperative pain scores.

### 3.1. SpO_2_, RR, and Haemodynamic Effects

**Figure 1:**
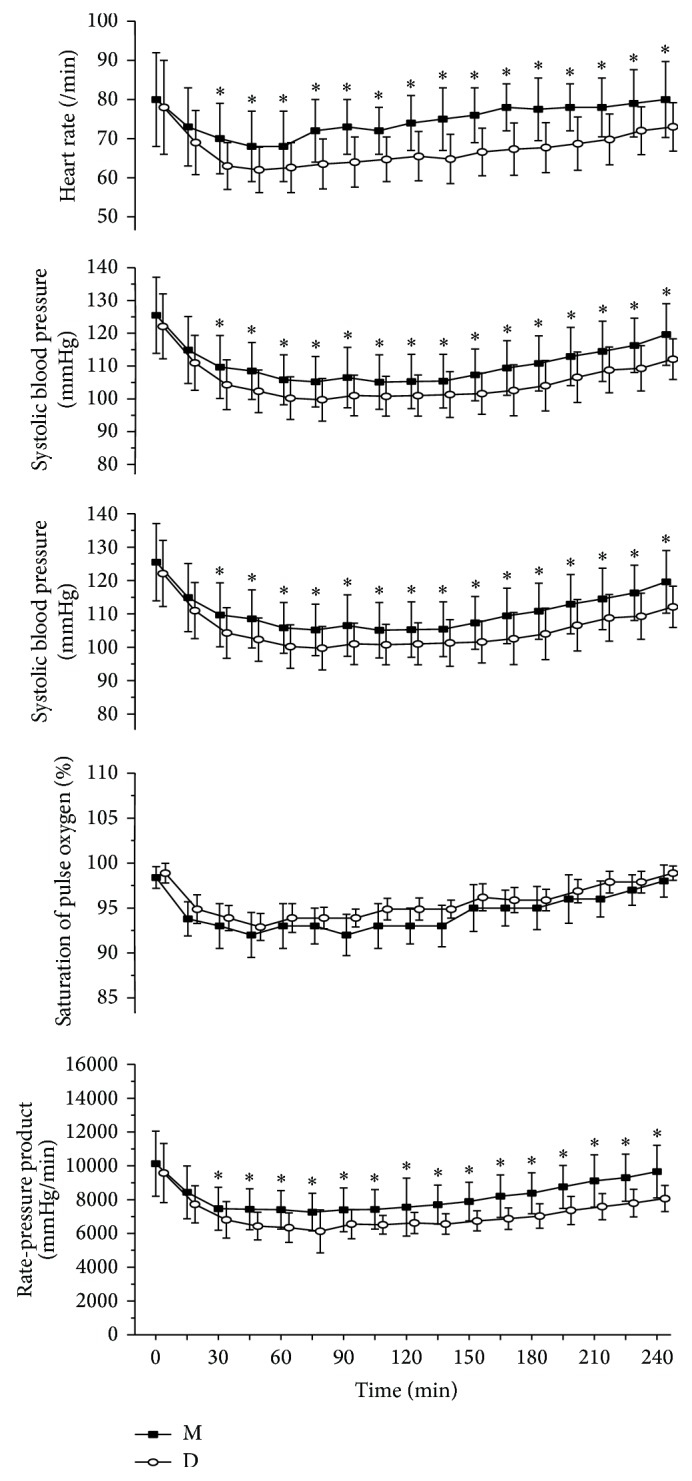
Heart rate, systolic blood pressure values, respiratory rate, saturation of pulse oxygen, and rate-pressure product (mean ± SD) for the two treatment groups (group M and group D) during the course of 240 min. Time 0 min = before drug administration. M: midazolam; D: dexmedetomidine. ^∗^ *P* < 0.05.

Figure 1 shows the mean SBP, HR, SpO_2_, RR, and RPP at different time points in each group. The SBP, HR, and RPP of group D became significantly lower than those of group M 30–45 min after drug administration and remained so for the rest of the study. There was no difference in SpO_2_ or RR between groups D and M.

### 3.2. Sedative and Analgesic Effects

**Figure 2:**
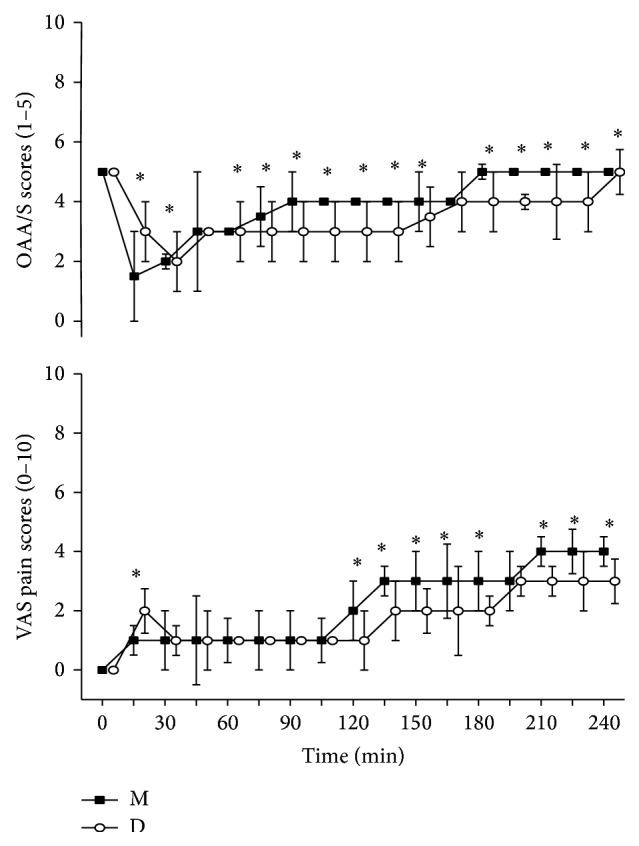
OAAS and VAS pain scores (median ± IQR) for the two treatment groups (group M and group D) during the course of 240 min. Time 0 min = before drug administration. M: midazolam; D: dexmedetomidine. ^∗^ *P* < 0.05.

Figure 2 graphically displays the median ± IQR. OAAS scores and VAS pain scores in both treatment groups were recorded at 15 min intervals until 4h after the start of drug infusion. The sedation level of group D became significantly different from that of group M 60–75 min after drug administration, and the differences remained statistically significant for the rest of the study period. The OAAS scores of group M were lower than that of group D 15–35 min after drug administration. Main reasons are as follows: the onset time of midazolam was 30~60 seconds and it takes 5 minutes to reach peak plasma drug concentration. However the onset time of DEX was 10~15 min and it takes 25~30  minutes to reach peak plasma drug concentration. The VAS pain scores in group D and group M were not statistically different after 30–120 min but became lower than that in group M after 120–240 min after drug administration. The pain is more intense as the local anesthetic wears off. DEX has analgesic effect but midazolam has not. Midazolam has shorter onset time relative to DEX, so the VAS pain score of group M was lower than that of group D 15 min after drug administration.

### 3.3. Anti-Inflammatory and Antioxidant Effects

**Figure 3:**
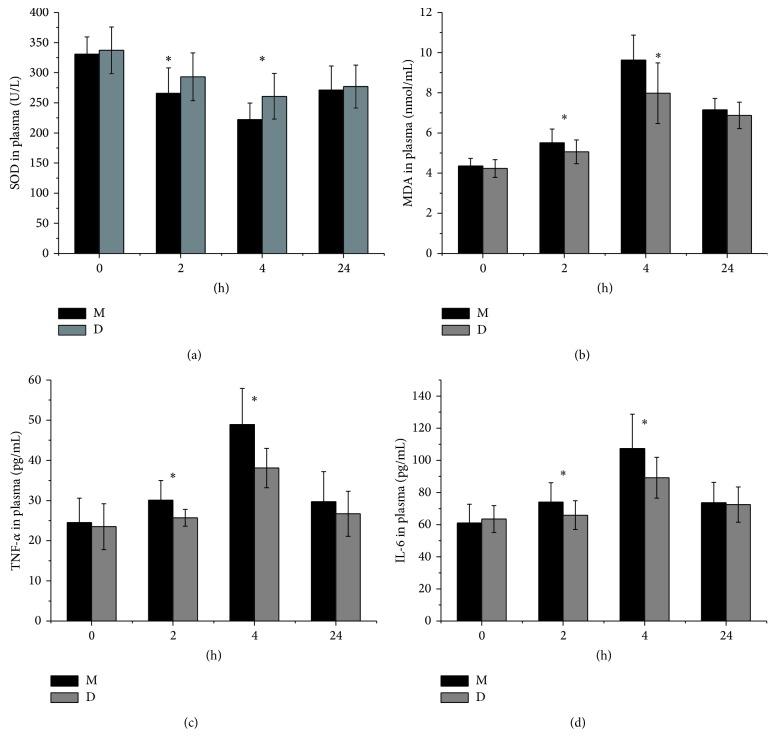
Plasma SOD, MDA, TNF-*α*, and IL-6 concentrations (mean ± SD) were investigated in each plasma sample for the two treatment groups (group M and group D). Time 0 h = before drug administration. Time 2 h = 2 h after drug administration. Time 4 h = 4 h after drug administration. Time 24 h = 24 h after drug administration. M: midazolam; D: dexmedetomidine. ^∗^ *P* < 0.05.

As shown in Figure 3, plasma SOD levels were not statistically different either at baseline (0 h) or at 24 h after drug administration in both groups. However, significant reduction of SOD was seen in group M but not in group D at 2, 4 h after drug administration (*P* < 0.05 group D versus group M) (Figure 3(a)), indicating that DEX prevented the reduction in plasma SOD levels. Similarly, plasma MDA level was not statistically different 0, 24 h after drug administration in both groups, while plasma MDA levels in group D were lower than group M 2, 4 h after drug administration (*P* < 0.05, Figure 3(b)). Plasma levels of IL-6 and TNF-*α* were lower in group D than those in group M at 2 and 4 h after drug administration (*P* < 0.05, *P* < 0.05, Figures 3(c) and 3(d)). Plasma TNF-*α* and IL-6 levels were not statistically different 0, 24 h after drug administration in both groups (Figures 3(c) and 3(d)).

### 3.4. Correlation Analysis

**Figure 4:**
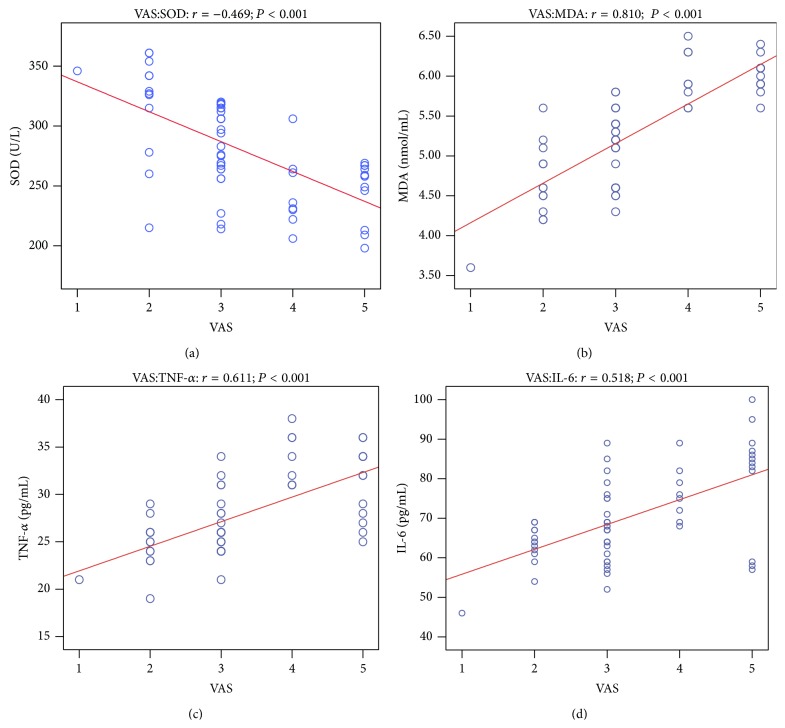
The correlation analysis between VAS pain scores and plasma concentrations of SOD, MDA, TNF-*α*, and IL-6 at 2 after drug administration. VAS pain scores versus SOD (a); VAS pain scores versus MDA (b); VAS pain scores versus TNF-*α* (c); VAS pain scores versus IL-6 (d).

**Figure 5:**
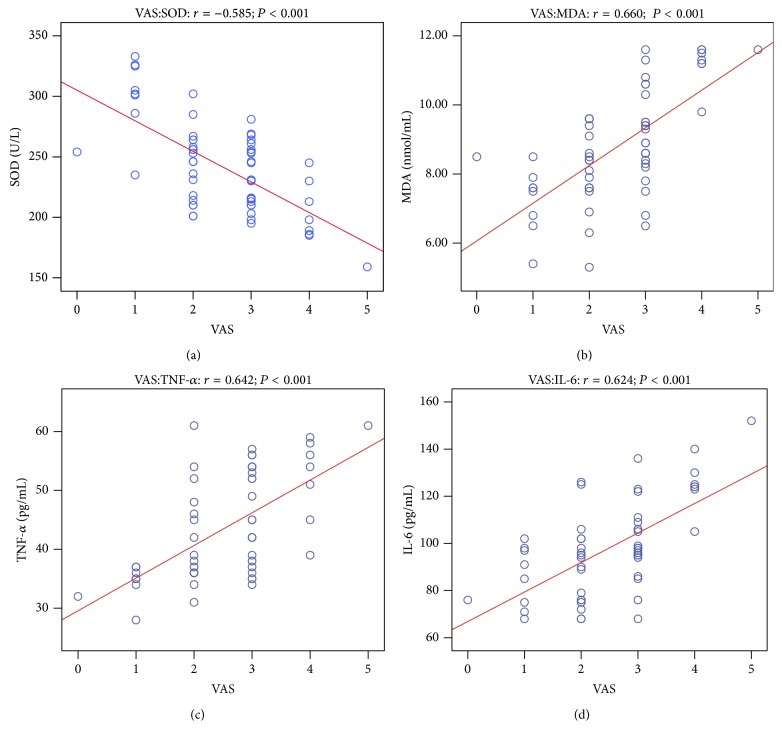
The correlation analysis between VAS pain scores and plasma concentrations of SOD, MDA, TNF-*α*, and IL-6 at 4 after drug administration. VAS pain scores versus SOD (a); VAS pain scores versus MDA (b); VAS pain scores versus TNF-*α* (c); VAS pain scores versus IL-6 (d).

**Figure 6:**
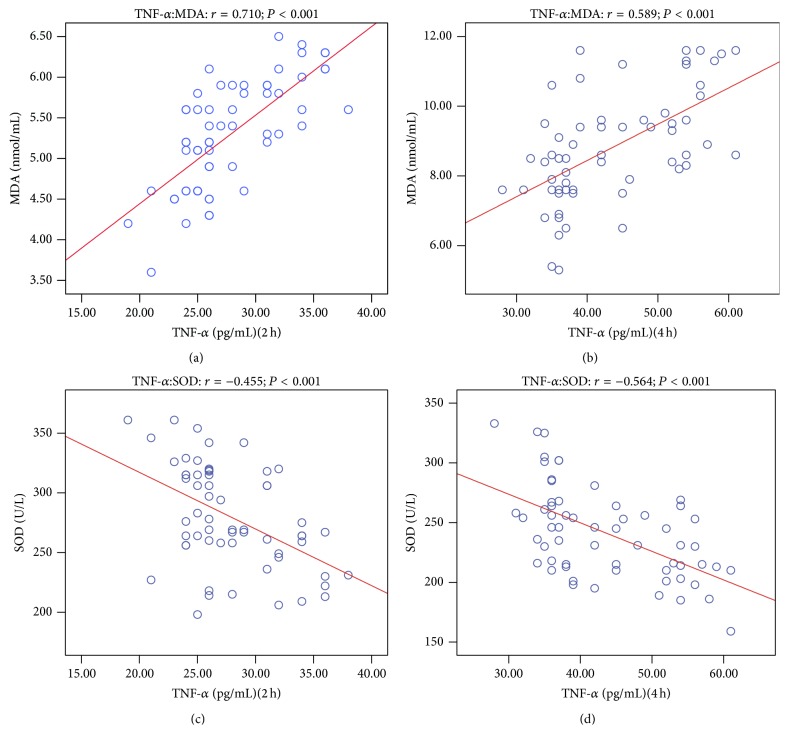
The correlation analysis between plasma concentrations of MDA and TNF-*α* after 2 h of drug administration (a); the correlation analysis between plasma concentrations of MDA and TNF-*α* after 4 h of drug administration (b); the correlation analysis between plasma concentrations of SOD and TNF-*α* after 2 h of drug administration (c); the correlation analysis between plasma concentrations of SOD and TNF-*α* after 4 h of drug administration (d).

We surmised that DEX offered better postoperative analgesia by regulating the inflammatory and oxidation factors. The correlation analyses between VAS pain scores and plasma concentrations of SOD, MDA, TNF-*α*, and IL-6 at 2, 4 after drug administration are shown in Table 2. Spearman analysis showed that VAS pain scores and plasma SOD content of the two groups were negatively correlated at 2, 4 h after drug administration. VAS pain scores and plasma MDA content were positively correlated. VAS pain scores were also positively correlated with plasma TNF-*α* and IL-6 content (Figures 4 and 5). Inflammation may contribute to oxidizing reaction. Our results showed that plasma TNF-*α* and SOD content of the two groups were negatively correlated, while plasma TNF-*α* and MDA content of the two groups were positively correlated (Figure 6).

## 4. Discussion

We have shown in the current study that DEX offered better sedation and postoperative analgesia on implant surgery compared with midazolam, which was associated with more pronounced reductions of postoperative plasma levels of TNF-*α*, IL-6, and MDA and an increase in SOD. The positive correlations between VAS and TNF-*α*, IL-6, and SOD provide evidence to suggest that DEX could offer better postoperative analgesia by suppressing inflammatory and oxidation response during implant surgery.

In addition to treatment for sedation and analgesia, the most significant adverse reactions associated with DEX are hypotension and bradycardia. DEX has been administered to hypertensive patients during surgery [24], suggesting a relaxing effect on peripheral vessels. Previous studies reported that RPP was one of the major determinants of myocardial oxygen consumption and RPP > 20,000 mmHg min^−1^ could precipitate angina pectoris [25]. No patient had an RPP of more than 20,000 mmHg min^−1^ in our study. The effect of DEX on lowering SBP, HR, and RPP could decrease the myocardial oxygen requirement and may be advantageous for patients at risk of coronary artery disease [26]. DEX can be titrated to the desired level of sedation without significant respiratory depression [27]. Midazolam often causes respiratory depression, especially with fentanyl or other opioids [28]. In the present study the SpO_2_ and RR did not differ significantly between the groups, despite the fact that the SBP and HR values slightly lower in group D. No incidence of cardiovascular instability that required intervention occurred in any of the patients.

Pain, inflammation, and postoperative trismus are the main symptoms following implant surgery. The pain is more intense from the first three to five hours as the local anesthetic wears off [29]. DEX can exert analgesic effects through activation of central *α*_2_-adrenergic receptors in the locus coeruleus [14]. In our study, VAS pain score was below about 4 in both groups during and after implant surgery. VAS pain score in group D was lower than group M during 120 min–240 min. OAAS scores of group D were lower than group M during 60 min–240 min. So, compared with midazolam, DEX offered better sedation and analgesia during and after implant surgery.

It is known that an increase in the level of proinflammatory cytokines, including TNF-*α* and IL-6, is an early feature of acute injury. Recent studies found that DEX has an anti-inflammatory effect by reducing the levels of inflammatory cytokines. A body of animal and clinical trials [30, 31] have shown that DEX decreases cytokine (TNF-*α*, IL-6) secretion after endotoxin injection and that DEX reduced the mortality rate in endotoxemia-induced shock rat models in a dose-dependent manner. In addition, several studies [32–34] have demonstrated that DEX could exert a potential protective effect by suppressing inflammatory responses on ventilator, lipopolysaccharide, or *α*-naphthylthiourea-induced acute lung injury. Compared to group M, our results showed that DEX exhibited potent activity in inhibiting TNF-*α* and IL-6 in dental surgery, particularly 4 h after drug administration. Although studies have shown the regulatory effects of DEX on inflammatory reactions, the exact mechanisms responsible for these actions are not well understood.

TNF-*α* is a major proinflammatory cytokine produced not only in the immune system but also in the peripheral and central nervous system, especially under the pathological conditions [35]. TNF-*α* is also known for its substantial role in periodontitis [36]. Increasing evidence suggests a critical role of TNF-*α* in the pathogenesis of pain including neuropathic pain [37, 38] and acute and persistent inflammatory pain [9, 39]. IL-6 induces muscle and joint hyperalgesia [40] and mediates the development of injury-induced hyperalgesia [41]. Following surgery, IL-6 levels are associated with postoperative pain [42]. In samples of patients with pain, levels of IL-6 have been shown to correlate with higher pain severity [43, 44]. Collectively, these findings support that proinflammatory cytokines are likely to play a facilitatory role in the development and maintenance of persistent pain syndromes. Our results showed VAS pain scores and plasma TNF-*α*, IL-6 content were positively correlated at 2, 4 h after drug administration. This suggests that postoperative pain may be caused by acute inflammation and that reducing inflammation cytokine release should have played an important role in DEX mediated reduction of postoperative pain in patients undergoing implant surgery.

SOD has strong antioxidant and physical activity and serves as a major free radical scavenger of body [45]. MDA, the end product of lipid peroxidation [46], was assessed in combination with SOD to evaluate the effects of DEX on oxidative stress during and after dental implant surgery in the current study. Some studies [22, 47, 48] have shown that DEX can attenuate the increase of MDA level and enhance SOD activities. In our study, the plasma MDA was higher in group M as compared to group D, while SOD activities were significantly lower in group M as compared to group D. These results pointed to possible antioxidant effects of DEX in the dental implant region. A few studies reported that various reactive oxygen species (ROS) scavengers and antioxidants reduce hyperalgesic behaviours in rat models of persistent pain [45]. Superoxide anion (O_2_^−^) is critical for sensitization of spinal neurons and persistent pain [49, 50]. Antioxidant enzyme SOD is concerned with the removal of superoxide anion. One study shows that saliva and serum antioxidants and serum MDA levels were elevated in patients with complex regional pain syndrome-type I [50]. Our results showed VAS pain scores and plasma SOD content of the two groups were negatively correlated. VAS pain scores and plasma MDA content were positively correlated. This suggests that postoperative pain may be caused in part by acute oxidative stress reaction. Thus, reduction of postoperative oxidative stress should also play an important role in DEX mediated attenuation of pain.

An exaggerated inflammatory response to tissue injury, ischemia, and reperfusion injuries can all result in excessive production of free radicals [51]. Free radicals, in turn, can increase vascular permeability, release neuropeptides (i.e., substance P), enhance inflammation, and cause further tissue damage [52, 53]. TNF-*α* increased the levels of superoxide anion and MDA and then induced oxidative stress and cell toxicity [54, 55]. A small dose of hydrogen peroxide enhances toxicity of TNF-*α* in inducing human vascular endothelial cell apoptosis [56]. Our result showed that plasma TNF-*α* and MDA, SOD content were correlated closely. While correlation relationship does not necessarily indicate a causal relationship, the findings of our current study provide mechanistic clues for future in-depth study to elucidate the mechanism of dexmedetomidine in clinical settings.

## 5. Conclusions

Our study demonstrates that DEX appears to provide better sedation, postoperative analgesia than traditional medicine midazolam during office-based artificial tooth implantation. Further, our findings provide evidence to suggest that reduction of postoperative inflammatory and oxidative stress plays important role in DEX postoperative analgesic effects, although detailed mechanism needs further study.

## Figures and Tables

**Table 1:** Clinical characteristics of patients for the two treatment groups, D and M.

Variables	Group D	Group M	*P* value
	(*n* = 30)	(*n* = 30)	
Age (year)	41.61 ± 9.82	43.34 ± 8.43	0.491
Body weight (kg)	61.12 ± 8.63	59.20 ± 7.73	0.384
Males/females	19/11	18/12	0.070
Duration of surgery (min)	60.16 ± 12.21	62.83 ± 10.72	0.372
Number of dental implants	2.53 ± 0.51	2.50 ± 0.51	0.800
Total volume of local anaesthetic used (mL)	1.72 ± 0.38	1.85 ± 0.43	0.232

M: midazolam; D: dexmedetomidine. Data shown are the number or mean ± standard deviation.

**Table 2:** The correlation analysis between VAS pain scores and plasma concentrations of SOD, MDA, TNF-*α*, and IL-6 at 2, 4 after drug administration.

Source	Dependent	Spearman	Sig.
	variable	correlation	(2-tailed)
VAS pain scores	SOD	−0.649^**^ (2 h)	<0.001
		−0.585^**^ (4 h)	<0.001
	MDA	0.810^**^ (2 h)	<0.001
		0.660^**^ (4 h)	<0.001
	TNF-*α*	0.611^**^ (2 h)	<0.001
		0.642^**^ (4 h)	<0.001
	IL-6	0.518^**^ (2 h)	<0.001
		0.624^**^ (4 h)	<0.001

^∗∗^Correlation is significant at the 0.01 level (2-tailed).
